# Impact of levels of total digestible nutrients on microbiome, enzyme profile and degradation of feeds in buffalo rumen

**DOI:** 10.1371/journal.pone.0172051

**Published:** 2017-02-16

**Authors:** Anju Kala, D. N. Kamra, Avinash Kumar, Neeta Agarwal, L. C. Chaudhary, C. G. Joshi

**Affiliations:** 1 ICAR National Professorial Chair, Center of Advanced Faculty Training in Animal Nutrition, Indian Veterinary Research Institute, Izatnagar, India; 2 Dept. of Biotechnology, College of Veterinary Science, Anand Agricultural University, Anand, Gujarat, India; Universidade de Aveiro, PORTUGAL

## Abstract

The present study was aimed at understanding a shift in rumen microbiome of buffaloes fed various levels of total digestible nutrients. To understand the process, the metagenomics of rumen microbes, *in vivo* and *in vitro* rumen fermentation studies were carried out. Three rumen fistulated adult male Murrah buffaloes were fed three isonitrogenous diets varying in total digestible nutrients (70, 85 and 100% of TDN requirement) in 3X3 switch over design. On dry matter basis, wheat straw/ roughage content were 81, 63 and 51% and that of maize grain was 8, 16 and 21% in three diets respectively. After 20 d of feeding, rumen liquor and rumen contents were sampled just before (0h) and 4h post feeding. *Ruminococcus flavefaciens* and *R*. *albus* (estimated with real time PCR) were higher in high roughage diets. The predominant phyla in all the three groups were Bacteroidetes, Firmicutes followed by Proteobacteria, Actinobacteria and Fibrobacteres. A core group of more than fifty rumen bacteria was present in all the animals with very little variations due to level of TDN. The most predominant bacterial genera reported in order of decreasing abundance were: *Prevotella*, *Bacteroides*, *Clostridium*, *Ruminococcus*, *Eubacterium*, *Parabacteroides*, *Fibrobacter*, *Butyrivibrio* etc. The higher diversity of the enyzmes families GH 23, GH 28, GH 39, GH 97, GH 106, and GH 127 (the enzymes active in fibre and starch degradation) were significantly higher on 100%TDN diet while CE 14 (required for the hydrolysis of bond between carbohydrate and lignin) was higher on low TDN (70%) diet, indicating ester bond cleavage was better in animals fed high roughage (wheat straw) diet.

## Introduction

In most of the tropical countries, the ruminants are fed on lignocellulosic crop residues like cereal straws, stovers, sugarcane bagasse, oil cakes with small quantities of cereal grain and green fodder as protein and energy sources. The bioconversion of these poorly utilizable energy sources lead to the formation of utilizable form of energy (short chain volatile fatty acids) by the concerted and synergistic activities of rumen microbiome (consisting of bacteria, protozoa, fungi, archaea and bacteriophages) [1]. The rumen fermentation is a complex process controlled by the constituent rumen microbiome and enzymes excreted by them, but these are poorly understood. Until recently, only a small group of enzymes and a very few fibre degrading bacterial groups representing only about 2% of total bacterial 16S rRNA [2] were reported to be responsible for fibre degradation in the rumen. But the recent studies based on real time PCR and meta-transcriptomic analysis have reported several hundred enzyme components which act collectively and synergistically upon complex fibrous feed material [3]. Hundreds of genes active in degradation of cellulose have been identified belonging to various CAZy families from the rumen carbohydrate active enzyme database (CAZyme database).

The energy sources (cellulose/hemicelluloses/pectin) are hetero-polymers of hexoses, pentoses and phenolic monomers which are inter-linked with different bonds in various feeds which change during plant growth [4,5]. Accordingly, the enzyme and microbial profiles required for this type of feed degradation might change by shifting grain based diet to roughage based diet [6]. The efficiency of feed degradation in the rumen is dictated by the presence of a pool of enzymes secreted by highly diversified rumen microbiome. To create a rumen microbial eco-system which is the most suitable for extracting maximum nutrients from lignocellulosic crop residues, we shall have to find out suitable answers to a few important questions like how the microbial community changes (qualitatively and quantitatively) on alteration in diet of animals or on supplementation of rumen modifiers. Does a core group of microbes exists essentially irrespective of diets offered to the animals and how much they are responsible for feed utilization. And finally, to find out ways to create an efficient and stable microbial eco-system which can optimally degrade such poor quality feeds. Advanced technique of metatranscriptomics, where mRNA from a microbial community is directly subjected to next generation sequencing is a holistic approach dealing with both the abundance of various microbial groups and the identification of the active genes involved in various metabolic processes combining taxonomic and functional analysis.

To find out solutions for some of the above questions, current study was conducted to examine the changing pattern in genes encoding carbohydrate active enzymes and rumen microbial community structure in buffaloes by feeding three diets varying in roughage (wheat straw) and concentrate feeds (a mixture of maize grain, soybean meal and wheat bran). To test the fibre degrading ability of rumen microbiome of these animals, *in vitro* gas production test (IVGPT) was conducted using rumen liquor as inoculum. To our knowledge it is first paper which is addressing correlation between rumen metagenome and its metabolites in buffaloes in comprehensive manner using both *in vivo* and *in vitro* approaches.

## Materials and methods

### Ethics statement

The buffaloes used in this experiment were housed at Animal Nutrition Sheds, Indian Veterinary Research Institute, Uttar Pradesh, India. The sheds are well ventilated with separate provision of offering feed and water. All procedures regarding animal handling and treatments in this study were approved by the Committee for the purpose of control and supervision on experiments on animals (CPCSEA, Ministry of Environment and Forest, GOI).

### *In vivo* fermentation

**Fig 1:**
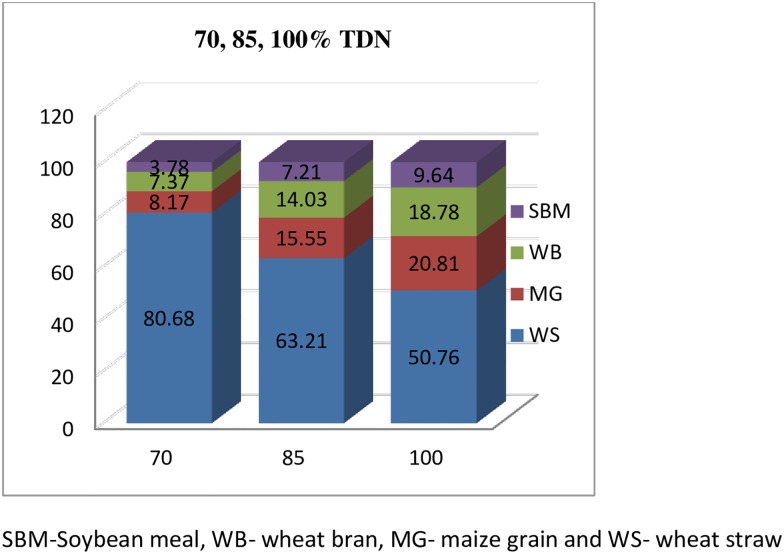
Composition of diet fed to buffaloes on dry matter basis.

In the first experiment, three fistulated male buffaloes (average BW 335±37.5 kg) were fed on diets containing 70, 85 and 100% of total digestible nutrients (TDN) of their requirement for maintenance as per ICAR feeding standards [7] in a Latin Square (Switch over) Design. The composition of the feed is depicted in Fig 1. The feed was offered at 9:00 h as a wet mixture of wheat straw and concentrate mixture (solid: water ratio 1:1). The rumen liquor and rumen content samples for microbial enumeration and enzyme estimation were collected at 0 and 4 hr post-feeding on two consecutive days. The samples were transported to the laboratory in an ice bucket and immediately processed and stored at -20°c.

### Enzyme estimation

The enzymes were extracted from the rumen content (5 g) with 25 ml phosphate buffer (0.1M, pH 6.8), 5 ml each of lysozyme (0.4%) and carbon tetrachloride [8]. The mixture was incubated at 39°C for 3 h, followed by freezing to stop reaction. The samples were centrifuged at 27000xg at 4°C for 15 min and the clear supernatant was used as a source of microbial enzymes. The activities of carboxymethylcellulase (CMCase), xylanase and amylase were estimated using carboxymethylcellulose, xylan and starch as substrate, respectively [9]. The assay mixtures were incubated for 60, 30 and 30 min at 39°C for CMCase, xylanase and amylase, respectively and the reducing sugars released were estimated [10]. The CMCase and amylase activities were expressed as nmol glucose produced ml^-1^ protein^-1^. Xylanase was expressed as nmol xylose produced ml^-1^ protein^-1^. For estimation of β-Glucosidase and, α-glucosidase activities, p-nitrophenyl-β-glucopyranoside and p-nitrophenyl-α-glucopyranoside [11] and for acetyl esterase, p-nitrophenol acetate [12] was used as substrate. The activity was defined as nmol p-nitrophenol released ml^-1^ protein^-1^. In the enzyme samples protein was estimated [13] and specific activity was presented as units/mg protein.

### Estimation of volatile fatty acids, ammonia nitrogen and lactic acid

For estimation of volatile fatty acids (VFA), 1 ml rumen liquor was mixed with 0.2 ml of 25% metaphosphoric acid and after 2 h, the samples were centrifuged at 5000xg for 10 min. The clear supernatant was injected into Chromosorb 101 column fitted in Gas Chromatograph equipped with Flame Ionization Detector, FID [14] with some modifications [15]. Sum of five volatile fatty acids (acetate, propionate, butyrate, iso-butyrate and valerate) was presented as total volatile fatty acids (TVFA). The rumen liquor was analysed for lactic acid [16] and ammonia nitrogen [17].

### Rumen microbial profile by real time PCR

The primer sequences and conditions used for real time PCR for various targeted microorganisms are listed in Table 1. Total genomic DNA was isolated by using Qiagen stool kit. For preparation of standard curve, the purified PCR product using specific primer was cloned in pGEMT easy vector (Promega) and transformed in *Escherichia coli*. The plasmid with insert was extracted and copy number was calculated. The plasmid was serially diluted to make standard curve and the copy number was calculated [18]. The amplification reactions were performed in a total volume of 20 μl, containing 2 ng of template DNA, 10 μl of 2X kappa SYBR master mix, 0.6 μl of each primer (10 μM) and nuclease free water to make up the volume to 20 μl.

**Table 1:** PCR primers for real time PCR assay with amplicon size and annealing temperature.

Target organism	Primer sequence (Forward/Reverse)	Size (bp)	Anneal temp. (°C)	Reference
Bacteria	F-5’CGG CAA CGA GCG CAA CCC-3’F-5’CCA TTG TAG CAC GTG TGT AGC C-3’	130	60	[19]
Fungi	F- 5’GAG GAA GTA AAA GTC GTA ACA AGG TTT -3’R-5’CCA AAT TCA CAA AGG GTA GGA TGAT T-3’	110	60	[19]
Methanogen	F 5’-TTC GGT GGA TCD CAR AGR GC-3’R 5’-GBA RGT CGW AWC CGT AGA ATC C-3’	140	60	[20]
*Ruminococcus albus*	F-5’CCCTAAACAGTCTTAGTTCG-3’R-5’CCT CCT TGC GGT TAG AAC A-3’	175	60	[21]
*R*. *flavefaciens*	F-5’CGA ACG GAG ATA ATT TGA GTT TAC TTA G-3’R-5’CGG TCT CTG TAT GTT ATG AGG TAT TA-3’	132	60	[19]
*Fibrobacter succinogenes*	F-5’GTT CGG AAT TAC TGG GCG TAA A-3’R-5’CGC CTG CCC CTG AAC TATC-3’	121	60	[19]

### RNA extraction and pyrosequencing

Rumen liquor sampled at 4 h post feeding on two consecutive days (~1 ml each day) was mixed with RNA later and stored for further use at –80°C. Total RNA was extracted from each sample and purified by RNeasy Mini Kit (Qiagen). Removal of ribosomal RNA was done by Ribo-Zero rRNA Removal Kit (Epicentre). Quality and quantity of mRNA was assessed on an RNA 6000 Pico Chip on the Agilent 2100 Bioanalyzer instrument. Total mRNA was fragmented and barcoded cDNA libraries were prepared for GS-FLx titanium platform according to manufacturer’s protocol. The emulsion-based clonal amplification (emPCR amplification) of cDNA libraries was carried out followed by sequencing by GS FLX Titanium (Roche, USA).

### Meta-transcriptome data analysis

The sequence data were preprocessed where sorting of sample wise sequences along with removal of adaptor sequences was performed. The sequences of low quality were removed using PRINSEQ perl script (http://prinseq.sourceforge.net). The reads for each of the samples were submitted to MG-RAST server (with default quality parameters) to analyze taxonomic and functional profiling of the meta-transcriptomic data. Total 473,159 high quality reads with an average of 52,573 reads per sample with length ranging from 369–395 bp and GC per cent ranging from 47–50 were obtained. Reads produced by the GS FLX Titanium were assembled using Newbler (version 2.3) and subjected to gene annotation on Carbohydrate-Active Enzyme Database (CAZy; http://www.cazy.org/) at cutoff value of 10^−5^. The data of rumen microbiome and CAZy were analysed by One way ANOVA of SPSS 16.0. The significant difference based on P-value, in the diversity of rumen microbiome of buffaloes as affected by the diets was also analysed by Analysis of Similarity (ANOSIM), a multivariate analysis with Bray-Curtis matrix using PAST (Paleontological Statistics) 3.0 software [22].

An *in vitro* gas production test was conducted using rumen liquor from above three animals as inocula on nine different feeds to achieve better correlation between *in vitro* results obtained and *in vivo* metabolite profile and rumen microbiome.

### *In vitro* gas production

The feed considered for *in vitro* fermentation were the feeds generally used in livestock feeding in Indian subcontinent. The feed tested were: hays (maize, oat and berseem), roughages (wheat straw, paddy straw and sugarcane bagasse) and diets comprising of ratios of concentrate mixture and wheat straw (20:80, 35:65 and 50:50). The concentrate mixtures were the same as described in the feeding trial. The feeds were dried and ground to pass 1 mm sieve. Exactly weighed feed (200 mg±10mg) was transferred in already calibrated syringes of 100 ml capacity in which 30 ml medium including 10 ml rumen liquor was dispensed anaerobically as per Menke and Steingass [23]. The syringes were incubated for 24h at 39°C. Each treatment was repeated in triplicate for two consecutive days.

### Estimation of gas and methane production

The incubation was terminated at 24h and displacement of piston in the syringes was recorded. The net gas produced in 24h was calculated by subtracting the reading of blank syringe (without substrate) from the value of test syringe. Methane production was estimated by injecting 100 μl gas from the head space of the syringe in the Porapak Q column of GC as described by Agarwal *et al*. [15].

### Estimation of *in vitro* true digestibility (IVTD)

After 24h incubation, the syringe contents were transferred in spoutless beaker by repeated washings with neutral detergent solution. The contents were refluxed for one hour and filtered through Gooch crucibles (Grade 1). The DM content of residue was weighed and IVTD was calculated as per the method described by Van Soest and Robertson [24].

### Statistical analyses

Metagenomic data was analysed applying PAST (Paleontological Statistics) 3.0 software and One way ANOVA. Data of feeding trial were analysed using General Liner Model Multivariate ANOVA with contrast analysis using the model, intercept + diet + period + diet x period to analyze the effect of diet, period and their interaction and for *in vitro* experiments the model, intercept + substrate+ inoculum + substrate x inoculum to analyze the effect of substrate, inoculum and their interaction. The means were compared using Tukey’s test if the main effect was significant (i.e., p<0.05) as per SPSS 16.0.

## Results

### Rumen fermentation and metabolites

The composition of diet and the post-feeding period affect pH of rumen content which was significantly higher at 85% TDN diet as compared to 70% TDN diet but was similar to 100% TDN diet irrespective of sampling time, however, pH dropped significantly after 4h post feeding as compared to 0h in all the three diets (Table 2). The activities of various enzymes like carboxymethylcellulase, xylanase, α-glucosidase, β-glucosidase, amylase and acetyl esterase were not affected either by diet or the post feeding period (Table 3). The diet composition did not affect the levels of total volatile fatty acids, acetate, propionate, butyrate, iso-butyrate and acetate: propionate ratio except valerate which was significantly higher (P<0.05) at 85% TDN diet as compared to 70% TDN diet but was similar to 100% TDN diet (Table 4). Lactic acid concentration in the rumen liquor of animals fed diet of 100% TDN (the highest level of maize grain) was significantly higher (P<0.05) as compared to the other two groups.

**Table 2:** Effect of diets varying in TDN content on rumen pH in buffaloes.

Period	TDN in diet (%)			Mean	SEM	P Value		
	70	85	100			Diet	Period	Diet*Period
0 hr	6.71	6.88	6.83	6.80^x^	0.03	0.02	0.001	0.65
4 hr	6.54	6.65	6.57	6.58^y^				
Mean	6.62^b^	6.76^a^	6.70^a^^b^					

^ab^Means with different superscripts in a row differ significantly (P<0.05);

^xy^ Means with different superscripts in a column differ significantly (P < 0.05);

Diet*Period- Interaction of diet and period

**Table 3:** Specific enzyme activity (Units/mg of protein) in buffaloes fed different levels of TDN.

Attributes	TDN in diet (%)			Mean	SEM	P Value		
	70	85	100			Diet	Period	Diet*period
CMCase								
0hr	72.7	47.6	55.0	58.4	6.4	0.50	0.10	0.30
4hr	70.7	72.8	86.3	76.6				
Mean	71.7	60.2	70.6					
Xylanase								
0hr	142	135	133	137	9.4	0.40	0.50	0.40
4hr	157	158	155	157				
Mean	150	147	144					
α-glucosidase								
0hr	1.9	2.4	2.4	2.2	0.2	0.90	0.31	0.73
4hr	2.0	2.5	2.3	2.3				
Mean	2.0	2.4	2.4					
β-glucosidase								
0hr	7.6	7.1	8.0	7.5	2.3	0.54	0.91	1.01
4hr	11.1	13.1	8.0	10.7				
Mean	9.3	10.1	8.0					
Acetyl esterase								
0hr	175	114	113	134	16.5	0.82	0.50	0.22
4hr	100	123	133	119				
Mean	138	118	123					
Amylase								
0hr	6.4	7.3	8.9	8.6	1.1	0.20	0.21	0.62
4hr	5.3	8.4	5.9	6.2				
Mean	5.9	7.9	7.4					

Diet*Period- Interaction of diet and period

**Table 4:** Effect of diets varying in TDN content on VFA (mmol/dl), Ammonia nitrogen (mg/dl) and lactic acid (mg/dl) in the rumen of buffaloes.

Attributes	TDN in diet (%)			Mean	SEM	P Value		
	70	85	100			Diet	Period	Diet*Period
Acetate								
0hr	3.65	3.67	2.96	3.43	0.27	0.40	0.64	0.77
4hr	3.10	3.60	3.05	3.25				
Mean	3.38	3.63	3.01					
Propionate								
0hr	0.84	0.80	0.57	0.74	0.07	0.22	0.82	0.82
4hr	0.75	0.76	0.64	0.72				
Mean	0.80	0.78	0.60					
Butyrate								
0hr	0.56	0.54	0.38	0.49	0.06	0.41	0.90	0.62
4hr	0.46	0.59	0.47	0.51				
Mean	0.51	0.56	0.43					
Iso butyrate								
0hr	0.05	0.05	0.04	0.05	0.01	0.16	0.80	0.68
4hr	0.07	0.04	0.03	0.05				
Mean	0.06	0.04	0.03					
Valerate								
0hr	0.05	0.08	0.04	0.06	0.01	0.04	0.54	0.18
4hr	0.04	0.08	0.08	0.07				
Mean	0.05^b^	0.08^a^	0.06^a^^b^					
TVFA								
0hr	5.17	5.16	4.01	4.78	0.40	0.36	0.77	0.76
4hr	4.45	5.09	4.29	4.61				
Mean	4.81	5.13	4.15					
A:P ratio								
0hr	4.6	4.88	5.24	4.91	0.15	0.07	0.17	0.83
4hr	4.23	4.76	4.82	4.61				
Mean	4.41	4.82	5.03					
Lactic acid								
0hr	1.09	0.63	2.1	1.27^x^	0.41	0.01	0.02	0.92
4hr	0.85	0.6	2.25	1.23^y^				
Mean	0.97^b^	0.61^b^	2.71^a^					
Ammonia nitrogen								
0hr	8.22	9.58	7.62	8.47	0.57	0.43	0.17	0.56
4hr	7.84	7.44	7.04	7.44				
Mean	8.03	8.51	7.33					

^ab^Means with different superscripts in a row differ significantly (P<0.05);

Diet*Period- Interaction of diet and period

### Rumen microbial profile

The population density of total bacteria, *F*. *succinogenes*, methanogens and fungi was similar in all the three diets, but the numbers of *R*. *flavefaciens* and *R*. *albus* were significantly lower (P < 0.05) in the rumen liquor of buffaloes fed on 100% TDN diet as compared to the animals fed 70% TDN diets. There was an effect of post-feeding time on some microbes as the population of R. *flavefaciens* and total fungi was significantly lower at 4h post feeding as compared to 0h feeding (Table 5).

**Table 5:** Effect of diets varying in TDN content on microbial profile (log number of cells/ml RL) in the rumen of buffaloes.

Period	TDN in diet (%)			Mean	SEM	P value		
	70	85	100			Diet	Period	Diet*Period
Bacteria								
0hr	10.35	10.69	10.30	10.44	0.18	0.65	0.37	0.67
4hr	10.37	10.24	10.15	10.25				
Mean	10.36	10.46	10.22					
*Fibrobacter succinogenes*								
0hr	8.27	8.06	8.00	8.11	0.17	0.11	0.14	0.61
4hr	8.25	7.65	7.55	7.82				
Mean	8.26	7.86	7.78					
*Ruminococcus albus*								
0hr	6.94	6.69	6.68	6.77	0.18	0.04	0.13	0.28
4hr	7.06	6.27	5.97	6.44				
Mean	7.00^a^	6.48^a^^b^	6.32^b^					
*Ruminococcus flavefaciens*								
0hr	7.45	7.17	6.98	7.20^x^	0.15	0.02	0.047	0.58
4hr	7.34	6.63	6.60	6.86^y^				
Mean	7.40^a^	6.90^a^^b^	6.79^b^					
Fungi								
0hr	6.99	7.22	6.75	6.98^x^	0.20	0.95	0.045	0.28
4hr	6.61	6.24	6.67	6.51^y^				
Mean	6.80	6.73	6.71					
Methanogens								
0hr	7.84	8.10	8.10	7.91	0.12	0.86	0.07	0.40
4hr	7.71	7.58	7.58	7.66				
Mean	7.77	7.84	7.75					

^ab^Means with different superscripts in a row differ significantly (P<0.05);

^xy^ Means with different superscripts in a column differ significantly (P < 0.05);

Diet*Period- Interaction of diet and period

### Meta-transcriptome profile of rumen

**Fig 2:**
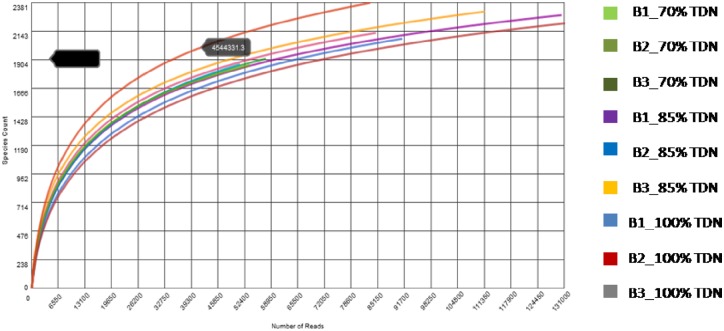
Individual rarefaction curve of metatranscriptomes of each sample of rumen liquor taken from buffaloes fed on different TDN (70, 85 and 100% of requirement) based diet.

**Fig 3:**
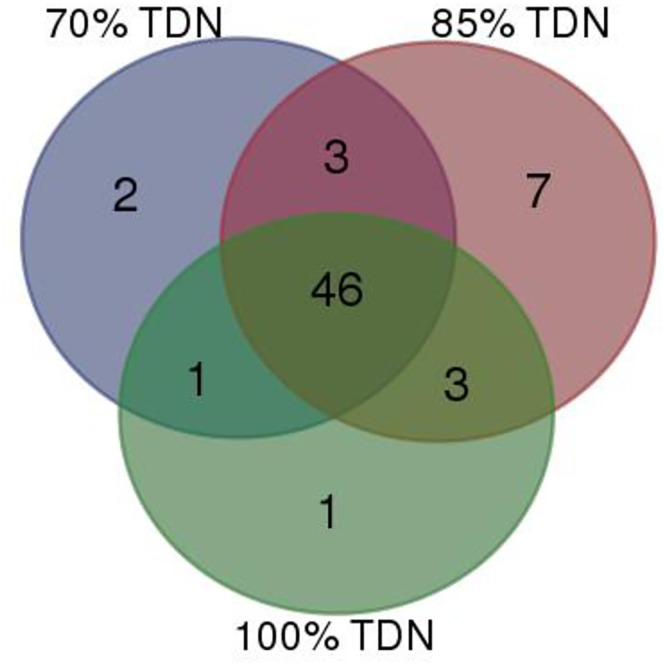
Shared phyla across different treatments. Venn plot showing shared and unique phyla in rumen of buffaloes fed 70, 85 and 100% TDN. All taxa present within each group are plotted.

**Fig 4:**
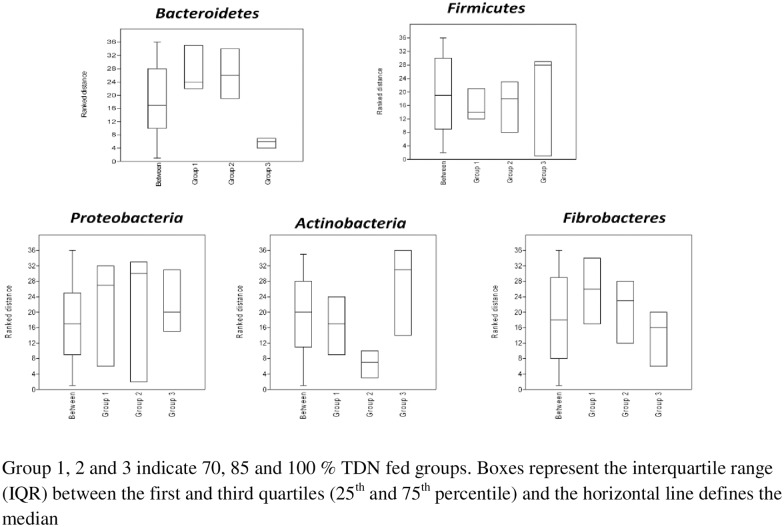
Box plot indicating mean of ranked distances for most abundant five rumen phyla in buffaloes fed three levels of TDN.

A total of 345 Mbp data was generated by pyrosequencing of nine samples from nine animals, three from each group. Rarefaction curve of metatranscriptomes of each sample of rumen liquor taken from buffaloes fed on different TDN (70, 85 and 100% of requirement) based diets is presented in Fig 2. The rarefaction curve (depicting an increase in bacterial species for increase in the number of sequencing reads) indicated that plateau was achieved and the sequences obtained in the experiment were sufficient to represent majority of the bacteria present in buffalo rumen. At domain level (S1 Table), bacteria were most abundant (78.56 to 84.15%) followed by eukaryota (12.68 to 6.99%). Archea contributed about 1.27 to 0.82% population; interestingly about 7% phyla abundance was of unassigned sequences. The taxonomic profiling of meta-transcriptome data showed that the total number of phyla obtained for each treatment (70%, 85% or 100% TDN) were 52, 59 and 51. The shared and unique phyla among the treatments were analyzed, where 46 phyla were found common in all the three treatments and unique phyla observed exclusively in 70%, 85% or 100% TDN were 2, 7 and 1, respectively (Fig 3), revealing not much variation among the three groups. Phylum level assignments of the data revealed Bacteroidetes and Firmicutes followed by Proteobacteria, Actinobacteria and Fibrobacteres as the predominant phyla in all the three groups. Interestingly, the order of abundance of these predominant phyla was similar for buffaloes of all the three groups except two animals, one from 85% TDN group and another from 100% TDN group where Fibrobacteres were higher than Actinobacteria. This might be simple individual animal variation. The abundance of Bacteroidetes and Firmicutes ranged from 31.69%-56.17% and 20.02%-23.15% representing an average of about 67 per cent of total population. ANOSIM results showed that there was not any significant difference (p<0.05) in the abundance of phyla with the change in diet (Fig 4). Dissimilarity (based on R values) was observed only for Bacteroidetes and Firmicutes but values did not differ statistically. Similarly, when ANOSIM was performed for some important bacterial genera like *Clostridium*, *Bacteroides*, *Ruminococcus*, *Eubactera*, *Parabeacteriodes*, *Fibrobacter* and *Butyrivibrio*, no difference was observed among the three treatments (P > 0.05). The F/B ratio was 0.5, 0.52, 0.48 in 70, 85 and 100% TDN diets, respectively, indicating some increasing trend with higher fibre in the diet. However, when analysed statistically (PAST 3.1), the three groups were similar. Eukaryota at phylum and genus level was observed to be contaminant sequences (probably through feed) as they were not of rumen origin and were not studied further.

Euryarchaeota was the major archeal phylum constituting about 90% of archeal population. In the rumen microbiome of buffaloes, *Methanobrevibacter* was the most predominant methanogen and *Methanoplanus*, *Methanoculleus*, *Methanospirillium*, *Methanothermobacter*, *Methanocorpusculum* were the other genera prevailing in the rumen of buffaloes (S3 Table).

**Fig 5:**
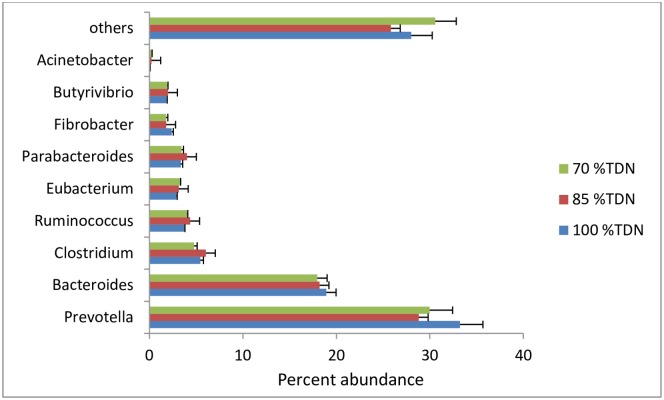
Per cent abundance of important bacteria in the rumen of buffaloes fed 70, 85, and 100% TDN of their requirement.

More than 50 genera of rumen bacteria were reported in all the animals with very small variations. The most important microbes reported in order of decreasing abundance were: *Prevotella*, *Bacteroides*, *Clostridium*, *Ruminococcus*, *Eubacterium*, *Parabacteroides*, *Fibrobacter*, *Butyrivibrio* etc and the per cent contribution of these bacteria were similar (P> 0.05) in all the three groups (Fig 5). By feeding diets varying in TDN content, there was no difference among the microbial phyla and genera, which could speak out about the effect of variable fibre content in the diet on the rumen microbial community structure.

### Diet and CAZyme distribution

**Fig 6:**
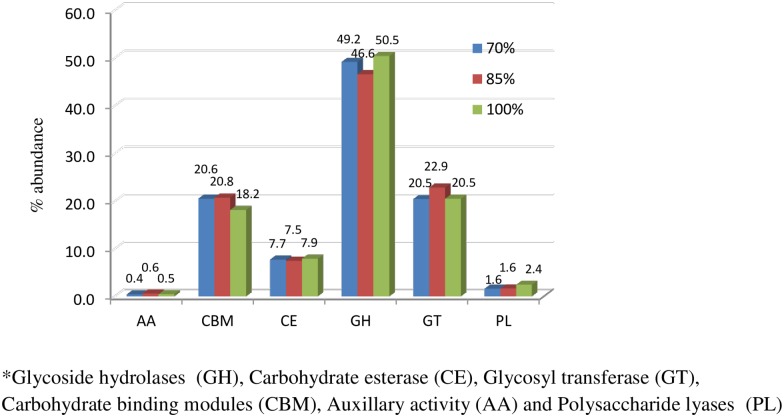
Per cent abundance of total CAZy classes in rumen of buffaloes fed various levels of TDN.

**Fig 7:**
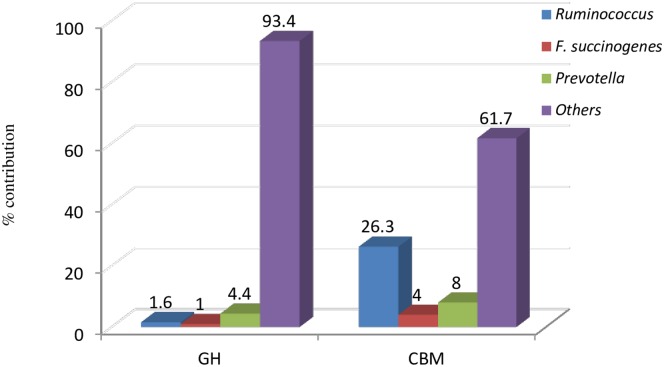
Per cent contribution of key cellulolytic bacteria in total GH and CBM CAZy families.

The meta-transcriptome data revealed a total of 41572 hits assigned to 89 CAZy families suggesting the expression of corresponding CAZy families in various treatments. The major CAZy classes including glycoside hydrolases (GHs), carbohydrate esterase (CEs), glycosyl transferase (GTs), carbohydrate binding modules (CBMs), auxillary activity (AA) and polysaccharide lyases (PLs) were represented by 20199, 3229, 8929, 8210, 213 and 792 hits, respectively. The average per cent of major CAZy classes was similar (P > 0.05) in all the three groups. Among all CAZymes, GHs was the highly expressed family (46.6–50.5%) followed by GTs (20.5–22.9%), CBMs (18.2–20.8%), CEs (7.5–7.9%), PLs (1.62–2.43%) and AA (0.4–0.6%) (Fig 6). The per cent abundance of each family was similar for all the three treatments except AAs and GHs, which represented significantly higher expression in 85% TDN diet. Though the overall abundance of families remained similar for all the three treatments, the abundance of enzymes belonging to respective families showed significant alterations with the treatments (S2 Table). In case of CBM family, CBM 35 was highly expressed enzyme in 100% TDN diet. CBM37, CBM48 and CBM50 represented 20.3–22.32, 14.16–15.74 and 13.81–17.19% of total CBMs showing no significant difference in three groups. The taxonomic annotations of the CBM families indicated that highly expressed enzyme CBM37 (456, 705 and 597 hits in 70, 85 and 100% diet, respectively) was contributed solely by *R*. *albus*. The sources of CBM48 and CBM50 included *Ruminococcus* sps., *F*. *succinogenes*, *P*. *ruminicola* but the overall contribution of known rumen bacteria towards CAZyme, especially GH and CBM was meager (Fig 7).

In case of GH families, few GHs like GH23, GH39, GH97, were significantly lower in 85% TDN diet as compared to other two diets, whereas, GH9 and GH127 were lower in 85% TDN diet than 70 and 100% TDN, respectively. GH106 was significantly lower in both 70 and 85% TDN diets as compared to 100% diet (S2 Table). Rest of the families of CAZy enzymes were not affected by any of the treatments. The GH2 and GH3 accounted for about 9 and 10% (the highest per cent) of the total GHs but were not represented by any of the known key fibre degrading bacteria like *F*. *succinogenes*, *R*. *flavefaciencs and R*. *albus*. GH45 represented very small fraction of total GHs and was solely contributed by *F*. *succinogenes*. In case of CE family, CE 14 was highly expressed enzyme in 70 and 85% TDN diets as compared to 100% TDN diet.

The overall contribution in the prime GH and CBM CAZy families of known microbes was less than 5%, however, there were large numbers of hits from *Ruminococcus*, rising up to 26.3% in CBM (Fig 7). The other two major contributors to CAZy familes reported in the study were *Clostridium* sps. and *Bacteroides* sps., but their representation was not so diversified as that of the above mentioned three key cellulolytic microbes. GH53 (a gene encoding an important hemicellulose degrading enzymes) was majorly represented by *Eubacteria* (upto 70%) followed by *Paenibacillus mucilaginosus*, *Amycolatopsis mediterranei* and *Paulibacter propionicigenes*.

### *In vitro* feed degradability by rumen microbiome

To test the efficacy of rumen microbiome of buffaloes fed on three different diets, *in vitro* fermentation of various feeds (three hays, three roughages and three complete diets) was conducted using rumen liquor of these animals fed the same diet as inocula. There was no effect of inocula (from 70, 85 and 100% TDN diet fed animals) on methane production expressed either as ml/g DM or ml/g DDM with different feeds and fodders except hays (Tables 6 and 7). In case of hays, methane production was significantly higher with inoculum from 100% TDN diet fed buffaloes irrespective of the type of hay. Among various substrates used, methane production was lower on paddy straw as compared to the other two roughage feeds (wheat straw and sugarcane bagasse), irrespective of inoculum used. There was no effect on total volatile fatty acids and acetate content when different feeds were fermented with buffalo rumen liquor from animals fed on different levels of TDN in diet (Table 8). Propionate level was significantly higher with inoculum of 70% TDN diet with roughages as substrate. Acetate: propionate ratio was significantly lower with inoculum from 70% TDN as compared to 100% TDN with roughages. When variable ratios of concentrate: wheat straw (diets) were used as substrate, the A: P ratio was significantly lower (P<0.05) with both 70 and 85% TDN diets as compared to 100% diet. The concentration of ammonia nitrogen was significantly higher (P < 0.01) in high TDN diets as compared to low TDN diets with various roughages and diets. Among hays, berseem hay produced significantly higher (P < 0.05) ammonia whereas values were similar for other substrates (Table 9).

**Table 6:** *In vitro* methane production (ml/gm DM) with rumen liquor of buffaloes fed different levels of TDN.

Substrate	Inoculum (% TDN in diet)			Mean	SEM	P Value		
	70	85	100			substrate	Inoculum	sub * Ino
Hay								
Maize hay	23.61	21.24	31.72	25.52	1.95	0.11	0.02	0.84
Oat hay	25.44	28.68	32.75	28.96				
Berseem hay	27.71	31.24	35.39	31.45				
Mean	25.59^b^	27.05^a^^b^	33.28^a^					
Roughage								
Wheat straw	19.41	23.74	24.17	22.44^x^	1.80	0.01	0.09	0.72
Paddy straw	12.94	14.75	17.78	15.16^y^				
Sugarcane bagasse	20.19	18.20	27.20	21.86^x^				
Mean	17.51	18.90	23.05					
Diet (concentrate: roughage ratio)								
20:80	26.28	26.05	29.94	27.42	2.18	0.08	0.47	0.98
35:65	32.04	33.22	34.42	33.22				
50:50	32.16	32.98	36.79	33.98				
Mean	30.16	30.75	33.72					

su ^ab^Means with different superscripts in row differ significantly (P<0.05);

^xy^ Means with different superscripts in column differ significantly (P < 0.05);

sub * Ino = substrate*inoculum interaction

**Table 7:** *In Vitro* methane production (ml/gm DDM) with rumen liquor of buffaloes fed different levels of TDN.

Substrate	Inoculum (% TDN in diet)			Mean	SEM	P Value		
	70	85	100			substrate	Inoculum	sub * Ino
Hay								
Maize hay	44.70	39.83	59.60	48.04	3.98	0.61	0.02	0.99
Oat hay	49.05	45.04	61.15	51.75				
Berseem hay	44.07	40.61	54.02	46.23				
Mean	45.94^a^^b^	41.83^b^	58.26^a^					
Roughage								
Wheat straw	41.94	55.12	63.81	53.62^x^	4.4	<0.001	0.24	0.32
Paddy straw	28.04	33.03	30.10	30.39^y^				
Sugarcane bagasse	57.85	44.06	63.73	55.21^x^				
Mean	42.61	44.07	54.15					
Diet (concentrate: roughage ratio)								
20:80	53.02	52.62	69.11	58.25	5.09	0.81	0.20	0.96
35:65	59.54	61.96	66.20	62.57				
50:50	55.66	55.57	67.05	59.43				
Mean	56.07	56.72	67.45					

su ^ab^Means with different superscripts in row differ significantly (P<0.05);

^xy^ Means with different superscripts in column differ significantly (P < 0.05);

sub * Ino = substrate*inoculum interaction

**Table 8:** Volatile fatty acids (mmol/dl) in different feeds incubated with rumen liquor of buffaloes fed various levels of TDN (70, 85 and 100%).

Substrate	Inoculum (% TDN in diet)			Mean	SEM	P value		
	70	85	100			Substrate	Inoculum	Sub*Ino
**TVFA**								
Hays								
Maize	5.71	4.55	3.29	4.52	0.52	0.61	0.06	0.9
Oat	4.09	4.66	2.96	3.9				
Berseem	4.53	4.08	2.91	3.84				
Mean	4.78	4.43	3.05					
Roughages								
Wheat straw	4.16	3.1	2.1	3.12	0.43	0.79	0.06	0.90
Paddy straw	3.67	3.71	2.22	3.2				
Sugarcane bagasse	3.82	3.73	3.0	3.52				
Mean	3.88	3.51	2.44					
Diet (concentrate: roughage ratio)								
20:80	3.88	3.51	2.44	3.28	0.45	0.99	0.49	0.89
35:65	3.71	3.74	3.64	3.7				
50:50	3.95	3.66	3.32	3.64				
Mean	4.07	3.58	3.31					
**Acetate**								
Hays								
Maize	4.43	3.44	2.6	3.49	0.43	0.65	0.10	0.94
Oat	3.22	3.43	2.34	3				
Berseem	3.56	3.05	2.35	2.99				
Mean	3.74	3.31	2.43					
Roughages								
Wheat straw	3.17	2.47	1.69	2.44	0.37	0.83	0.12	0.93
Paddy straw	2.87	2.88	1.77	2.5				
Sugarcane bagasse	2.88	2.99	2.36	2.74				
Mean	2.97	2.78	1.94					
Diet (concentrate: roughage ratio)								
20:80	2.83	2.94	2.93	2.9	0.38	0.96	0.75	0.94
35:65	2.92	2.85	2.57	2.78				
50:50	3.32	2.58	2.34	2.75				
Mean	3.02	2.79	2.62					
**Propionate**								
Hays								
Maize	1.21	0.98	0.74	0.98	0.14	0.77	0.10	0.93
Oat	0.94	1.14	0.63	0.9				
Berseem	0.94	0.96	0.62	0.84				
Mean	1.03	1.03	0.66					
Roughages								
Wheat straw	0.95	0.65	0.48	0.7	0.1	0.95	0.04	0.81
Paddy straw	0.79	0.85	0.45	0.69				
Sugarcane bagasse	0.86	0.73	0.61	0.73				
Mean	0.87^a^	0.74^a^^b^	0.51^b^					
Diet (concentrate: roughage ratio)								
20:80	0.83	0.8	0.62	0.75	0.11	0.90	0.09	0.84
35:65	0.92	0.76	0.65	0.78				
50:50	1.14	0.73	0.59	0.82				
Mean	0.96	0.76	0.62					
**Butyrate**								
Hays								
Maize	0.37	0.44	0.15	0.32	0.04	0.60	<0.001	0.40
Oat	0.18	0.42	0.23	0.27				
Berseem	0.25	0.34	0.2	0.26				
Mean	0.27^a^^b^	0.40^a^	0.19^b^					
Roughages								
Wheat straw	0.25	0.19	0.18	0.21	0.03	0.10	0.39	0.74
Paddy straw	0.20	0.2	0.09	0.17				
Sugarcane bagasse	0.29	0.24	0.28	0.27				
Mean	0.25	0.21	0.19					
Diet (concentrate: roughage ratio)								
20:80	0.26	0.25	0.29	0.27	0.04	0.35	0.43	0.98
35:65	0.36	0.29	0.36	0.34				
50:50	0.38	0.28	0.38	0.35				
Mean	0.34	0.27	0.35					
**A: P**								
Hays								
Maize	3.80	3.53	3.94	3.76	0.24	0.73	0.55	0.78
Oat	3.99	3.38	4.22	3.86				
Berseem	3.90	4.16	4.01	4.02				
Mean	3.89	3.69	4.06					
Roughages								
Wheat straw	3.45	4.24	3.87	3.86	0.22	0.77	0.04	0.47
Paddy straw	3.63	3.59	4.36	3.86				
Sugarcane bagasse	3.36	4.19	4.62	4.05				
Mean	3.48^b^	4.01^a^^b^	4.28^a^					
su Diet (concentrate: roughage ratio)								
20:80	3.45	3.65	5.72	4.27	0.21	0.15	<0.001	0.39
35:65	3.17	3.75	4.61	3.84				
50:50	2.92	3.73	4.51	3.72				
Mean	3.18^b^	3.71^b^	4.95^a^					

^ab^Means with different superscripts in row differ significantly (P<0.05);

sub * Ino = substrate*inoculum interaction

**Table 9:** *In vitro* ammonia nitrogen (mg/dl) in different feeds incubated with rumen liquor of buffaloes fed different levels of TDN.

Substrate	Inoculum (% TDN in diet)			Mean	SEM	P Value		
	70	85	100			Substrate	Inoculum	Sub*Ino
Hays								
Maize	6.29	5.60	9.08	6.99^x^^y^	0.62	0.04	0.07	0.74
Oat	6.64	5.93	7.26	6.61^y^				
Berseem	9.21	7.95	9.36	8.84^x^				
Mean	7.38	6.49	8.56					
Roughages								
Wheat straw	5.95	7.41	9.81	7.72	0.77	0.11	0.01	0.85
Paddy straw	7.92	8.28	11.81	9.34				
Sugarcane bagasse	5.48	7.61	8.17	7.09				
Mean	6.45^b^	7.77^a^^b^	9.93^a^					
Diets (Concentrate: roughage)								
20:80	7.77	6.02	11.30	8.36	0.63	0.66	0.001	0.45
35:65	7.57	8.51	11.01	9.03				
50:50	5.94	8.07	10.90	8.30				
Mean	7.10^b^	7.54^b^	11.07^a^					

^ab^Means with different superscripts in row differ significantly (P<0.05);

^xy^ Means with different superscripts in column differ significantly (P < 0.05);

sub * Ino = substrate*inoculum interaction

There was no effect of inocula collected from buffaloes fed on diets with variable TDN on IVTD of various feeds tested. Among the hays IVTD of berseem was significantly higher as compared to maize and oat, irrespective of inoculum type. Among the three diets (comprising of variable concentrate and roughage ratios) used as substrate, the IVTD increased with the increasing level of concentrate in the diet irrespective of the inoculum (Table 10).

**Table 10:** *In vitro* true digestibility (IVTD %) of different feeds with rumen liquor of buffaloes fed different levels of TDN.

Substrate	Inoculum (% TDN in diet)			Mean	SEM	P Value		
	70	85	100			substrate	Inoculum	sub * Ino
Hay								
Maize hay	55.57	52.61	51.91	53.36^y^	1.39	<0.001	0.30	0.57
Oat hay	57.06	62.76	55.31	58.38^y^				
Berseem hay	64.17	74.65	68.62	69.15^x^				
Mean	58.93	63.34	58.61					
Roughage								
Wheat straw	46.38	43.62	39.35	44.2	1.7	0.10	0.51	0.23
Paddy straw	47.81	45.18	47.45	44.92				
Sugarcane bagasse	38.45	45.98	41.57	43.66				
Mean	42.83	46.81	42.09					
Diet (concentrate: roughage ratio)								
20:80	38.78	46.75	44.56	43.36^y^	1.33	<0.001	0.52	0.79
35:65	54.01	55.69	55.74	55.15^x^				
50:50	60.73	62.2	57.41	60.11^x^				
Mean	51.17	54.88	52.57					

^xy^ Means with different superscripts in column differ significantly (P < 0.05);

sub * Ino = substrate*inoculum interaction

## Discussion

The microbiome of the rumen plays an important role in feed digestion and its bioconversion to utilizable energy sources (volatile fatty acids) and ultimately to edible or other useful livestock products like meat, milk wool etc. [25]. The microbial and enzyme profiles of the rumen have been difficult to identify in their entirety because of the limitations of conventional techniques of cultivation of rumen microbes [26], but the invention of molecular techniques like real time PCR and next generation sequencing have made the job easier.

The analysis of metagenomic libraries of rumen microbiome of buffaloes fed on diets of variable TDN contents (70, 85 and 100%) depicted no change in rumen microbial community which might be due to very narrow variation in the level of fiber in three diets. In our experimental diets, the maize grain varied from about 8.0 to 20.0 per cent and wheat straw varied was from 80.0 to 51.0 per cent of diet which might not be sufficient to alter rumen microbial consortia significantly. At phylum level, Bacteroidetes, Firmicutes, Proteobacteria, Actinobacteria and Fibrobacteres were the major phyla and the dietary changes could not affect their predominance. These results resembled several other researchers’ findings of rumen microbiome [27, 28]. The representation of Bacteroidetes and Firmicutes is comparatively lower than as reported earlier [29, 28] but lot of variations in the abundance of bacteroidetes and firmicutes among the animals has been reported by Jami *et al*. [30]. According to Li *et al*. [31], the rumen bacterial structure varies with the sampling time and location of rumen from where the sample has been collected. No change was observed in the abundance of Bacteroidetes, Firmicutes and Proteobacteria in the steers fed on diet supplemented with 2% of nitrate [32]. Several workers have reported the abundance of Bacteroidetes and Firmicutes in bovine rumen [33–36] as well as ovine rumen [37].

In the present study, the the F/B ratio was higher in higher fibre diet (low TDN diet). Similarly, a higher F/B ratio was observed in hay-fed group than high-concentrate-fed group using terminal restriction fragment length polymorphism (T-RFLP) analysis in four ruminally cannulated beef steers. This higher F/B ratio indicated higher fibre utilization which was supposed to be due to increase in the population density of *Ruminococcus flavefaciens* [29]. Similarly, in our study, a numerically higher F/B ratio and higher *Ruminococcus* count (by real time PCR) was observed with high fiber diet. A change in F/B ratio was also observed on introduction of dried distiller’s grains plus soluble in the diet of cattle previously fed feedlot ration [32].

The majority of bacterial genera reported were able to degrade lignocellulosic feeds. *Prevotella* was the predominant bacterium representing about 30 per cent of the total rumen bacteria. *Ruminococcus* and *Fibrobacter* were only 2–3 per cent of rumen bacterial community of buffaloes irrespective of the diet. Predominance of *Prevotella* and very little representation of *Ruminococcus* and *Fibrobacter* were also found in the goat rumen microbiome [38]. The already known three most important key fibrolytic bacteria viz, *R*. *flavefaciens*, *R*. *albus* and *F*. *succinogenes* represented only ~2% of ruminal bacterial 16S rRNA [2]. Very little representation of these fibrolytic bacteria might be the reason for no impact of diet variation on these microbes in most of the studies.

The CAZyme database deals with abundance of the sequences encoding for carbohydrate utilizing enzymes mainly glycoside hydrolases (GHs), which hydrolyze glycosidic bonds of carbohydrates, cellulose binding module (CBMs), involved in enzyme substrate binding and carbohydrate esterases (CEs), which break the ester bonds between lignin and carbohydrates. CAZyme profile depicted no difference in the metatranscriptomic libraries of rumen microbiome of buffaloes fed on diets differing in TDN levels. The auxiliary activities (AAs), which include lignolytic enzymes were significantly higher in the animals of 85% TDN diets. The GHs (52) and CBMs (22) were the most diversified as compared to other families. CBMs play a central role in plant polysaccharides hydrolysis being associated with the enzymes active in hydrolysis of plant cell wall. CBM37 can bind with homologous substrates like xylan, chitin, amorphous and crystalline cellulose and also with heterologous substrates like plant cell walls. CBM predominancy was higher in high fiber groups indicating that they have a major role to play in this process. Also the highest contribution of CBM came from CBM 37 which was solely contributed by *Ruminococcus*, a fibrolytic rumen microbe, further strengthening above hypothesis. Considering 100% TDN diet as standard, there were shifts in GHs (9, 23, 28, 39, 72, 97, 106 and 127) in the other two diets as compared to 100% diet, rest of the GHs were similar in all the three diets indicating not much impact of TDN levels on the diversity as well as abundance of various GH families (S2 Table). The nine GH families (GH8, GH10, GH11, GH26, GH28, GH51, GH53, GH67 and GH78) reported as some of the important proteins involved in hemicellulose degradation [3] were also present in metatranscriptomes of rumen microbiome of all the three buffaloes fed on three different TDN diets though GH48 could not be identified in any of the samples of buffaloes. These important exo-proteins are closely associated with cellulosomes and were barely identified in previous metagenomic libraries [39, 40 and 3]. It needs to be investigated that in the absence of these important proteins, how plant cell wall is degraded. The alternate possibility is that there might be some additional GH proteins other than GH48 which might be closely associated with cellulosomes and are responsible for plant cell wall degradation [3]. The abundance of families GH23 (lysozyme type G, peptidoglycan lyase and chitinase), GH28 (polygalacturonase, rhamnogalacturonase, α-1,2-galacturonohydrolase, endo-xylogalacturonan hydrolase), GH39 (β-xylosidase), GH97 (glucoamylase, α-glucosidase and α-galactosidase), GH106 (α-L-rhamnosidase) and GH127 (β-L-arabinofuranosidase) were more on normal (100%) TDN diet while CE 14 (carbohydrate esterase) was higher on high fiber/low TDN (70%) diet, indicating ester bond cleavage was better in animals fed high roughage (wheat straw) diet and amylose degrading enzymes were more on normal TDN (100%) diet. Increase in abundance of CE14 by increasing fiber level in the diet gives an indication that since these proteins are involved in detachment of lignin from the polysaccharides, therefore by increasing the fibre, the requirement for such proteins might increase to hydrolyze bond between lignin and carbohydrate, the abundance of such CAZyme family might fulfill the increased demand for such enzymes.

As reported earlier, the key role in fiber degradation is played by two major rumen bacterial genera represented by three species namely *Ruminococcus albus*, *R*. *falvefaciens* and *F*. *succinogenes* [41]. In the present study, the population of two major cellulolytic bacterial species i.e. *R*. *flavefaciens* and *R*. *albus* significantly decreased with reduction in wheat straw content in the diet, but the third important species i.e. *F*. *succinogenes* population was not altered on changing TDN level in diet, as assessed by real time PCR. A positive correlation between *Ruminococcus* species and fiber content of diet indicated their role as key fiber utilizers in rumen. *F*. *succinogenes* was the highest in number followed by *R*. *flavefaciens* and *R*. *albus*, irrespective of diet, when assessed by real time PCR. Similar pattern of distribution was also reported earlier [41]. Koike and Kobayashi [21] also did not find any change in the population of these three major cellulolytic bacteria with changing hay levels in the diet.

With the increase in TDN level in diet there was a significant increase in lactic acid concentration and this might be the reason of decreased population of fibrolytic bacteria (*Ruminococcus*) at high level of TDN. Similarly, in an experiment with goats, bacterial and chemical changes in the rumen were studied by stepwise adaptation of the animals to concentrate levels of 0, 30, 50 and 70% in diet. Up to 50% level of concentrate, the microbial diversity was not affected, but concentrate levels higher than that caused lactic acid accumulation and most of the fibrolytic bacteria were eliminated but *Streptococcus bovis* and *Prevotella* persisted in the rumen [42]. However, in present study, the increase in level of maize grain could not enhance the concentration of lactic acid up to a level that could influence ruminal pH, which is one of the most important factors, because the fibrolytic bacteria are very sensitive to low pH [43]. In archea, Euryarcheota was the most predominant phylum comprising of major methane producing archea and *Methanobrevibacter* was the most abundant genus in the rumen of all buffaloes. Also, the community structure of methanogen archaea was not influenced by TDN level in the diet. Predomimancy of *Methanobrevibacter* in archaeal community has been reported in majority of the rumen studies [44, 45] with little influence of diet on methanogen community [45]. It can be said that the diet neither changed methanogen diversity in buffaloe rumen nor could affect *in vitro* methane production. Eukaryota sequences obtained were not of rumen origin and thus were not studied further. In our study, we did not expect rumen fungi or protozoa sequence (eukaryota) to amplify as metatranscriptomic sequencing was done by 16S rRNA.

Lignocellulosic feed ingredients like wheat straw, paddy straw, sugarcane by-products are not completely utilized by the rumen microbes as about 20–30% of the potential energy of these feeds is excreted undigested from the body of the animal. Depending on the structure of lignocellulosic feeds, a definite microbial consortium and a pool of diversified enzymes are needed for extraction of additional nutrients from such feeds and modulate other physiological processes [30] which might result into improved extraction of nutrients and livestock productivity.

The knowledge of metatranscriptomics of rumen microbiome on a defined feeding regimen can provide information about the type of microbial or enzyme consortia required for the digestion of a specific type of feed. Therefore, efforts have been made to find out a correlation between diet composition and microbial diversity and enzyme profile to find out the most suitable consortium of microbes which might be responsible for optimum degradation of lignocellulosic feeds. For this *in vitro* experiments were conducted using various feeds as substrate and rumen liquor from the buffaloes fed on diet with 70, 85 and 100% TDN was used as inocula. Similar rumen microbiome in the buffaloes of all the three groups might be the reason for similar gas, methane, and VFA production and IVTD with the inocula of the buffaloes fed different diets. However, ammonia nitrogen was higher with inoculum from the buffaloes fed 100% TDN diet (with roughages and diets as substrates) indicating that utilization of rumen nitrogen was more efficient at lower TDN levels., as all buffaloes were fed isonitrogenous diets.

## Conclusions

The study was aimed to understand shift in rumen microbiome of buffalo fed various levels of TDN and to correlate it with *in vivo* fermetation and *in vitro* feed digestibility so as to achieve a complete knowledge about buffalo rumen microbiome fed feeds differing in TDN content. A higher F/B ratio (estimated by metagenomics) in high fiber groups was positively correlated with higher *Ruminococcus* population (by real time PCR) in these groups indicates that these microbes are essentially required for fiber degradation. Only a very few parameters differed among the treatments, which might not be sufficient to clearly define the fibre degrading ability of rumen microbiome. When the rumen liquor from differently fed buffaloes, as described above, was used as inoculum in *in vitro* gas production test, a difference was observed in methane production when different hays were used as substrate, but there was no effect on feed digestibility and volatile fatty acids production. These results reveal a complex microbial diversity in the rumen of buffaloes with no effect of diet on profile of microorganisms involved in degradation of polysaccharides and gene pool of carbohydrate active enzymes. The variations in concentrate mixture (19–49% of the ration) or maize grain content (8–21% in the concentrate mixture) in the diets were not sufficiently enough to express change of appreciable magnitude in the microbial diversity or enzyme profile of rumen. Therefore, it appears that wider feed variations, especially in grain content are required to bring out significant shifts in metatranscriptomic libraries so that contributions of genes of interest derived from specific microbes for cellulose degradation could be identified which might be further targeted for rumen manipulation to maximize extraction of nutrients from poor quality lignocellulosic feed. One more thing observed was that even after feeding of similar diet, individual variation of animal plays important role in deciding rumen microbiome of ruminant.

## Supporting information

S1 Table Per cent abundance of buffalo rumen microbiome domain on various levels of TDN (%). (DOCX)

S2 Table Effect of TDN in diet on CAZY families’ abundance of buffalo rumen. (DOCX)

S3 Table Abundance (range) of archeal genera in rumen microbiome of buffaloes. (DOC)
