# Supplementary material for: Impact of levels of total digestible nutrients on microbiome, enzyme profile and degradation of feeds in buffalo rumen
Source: PLoS One. 2017 Feb 16;12(2):e0172051. doi: 10.1371/journal.pone.0172051 (PMC5313230; doi:10.1371/journal.pone.0172051)
Supplement: S1 Table — (DOCX) [file pone.0172051.s001.docx]

S1Table. Percent abundance of buffalo rumen microbiome doamin on various levels of TDN (%)

| domain | % abundance | | |
| --- | --- | --- | --- |
|  | 70 % | 80 % | 100 % |
| Archaea | 1.27 | 1.25 | 0.82 |
| Bacteria | 79.13 | 78.56 | 84.51 |
| Eukaryota | 12.01 | 12.68 | 6.99 |
| Viruses | 0.05 | 0.05 | 0.05 |
| other sequences | 0.02 | 0.01 | 0.01 |
| unassigned | 7.31 | 7.28 | 7.38 |
| unclassified sequences | 0.23 | 0.17 | 0.25 |
