# Supplementary material for: Impact of levels of total digestible nutrients on microbiome, enzyme profile and degradation of feeds in buffalo rumen
Source: PLoS One. 2017 Feb 16;12(2):e0172051. doi: 10.1371/journal.pone.0172051 (PMC5313230; doi:10.1371/journal.pone.0172051)
Supplement: S2 Table — (DOCX) [file pone.0172051.s002.docx]

S2 Table. Effect of various levels of TDN in diet on CAZY families’ abundance of buffalo rumen

| CAZy families | Diets with different levels of TDN (%) | | | SEM | P Value |
| --- | --- | --- | --- | --- | --- |
|  | 70 | 85 | 100 |  |  |
| CBM2 | 4.47 | 5.04 | 4.91 | 0.421 | 0.879 |
| CBM3 | 1.06 | 0.88 | 0.53 | 0.147 | 0.368 |
| CBM4 | 1.03 | 0.8 | 1.18 | 0.213 | 0.805 |
| CBM5 | 1.24 | 0.68 | 0.84 | 0.132 | 0.218 |
| CBM6 | 9.08 | 8.31 | 10.05 | 0.653 | 0.616 |
| CBM9 | 1.58 | 1.45 | 1.33 | 0.178 | 0.883 |
| CBM12 | 1.88 | 2.82 | 1.31 | 0.352 | 0.226 |
| CBM13 | 5 | 4.46 | 5.37 | 0.459 | 0.774 |
| CBM20 | 1.73 | 3.36 | 3.24 | 0.419 | 0.223 |
| CBM22 | 0.76 | 0.41 | 0.6 | 0.169 | 0.755 |
| CBM26 | 0.47 | 1.16 | 0.22 | 0.282 | 0.425 |
| CBM27 | 1.25 | 0.88 | 1.36 | 0.168 | 0.536 |
| CBM32 | 6.72 | 6.86 | 7.09 | 0.689 | 0.982 |
| CBM35 | 1.59^a^ | 1.72^a^ | 4.50^b^ | 0.505 | 0.002 |
| CBM37 | 21.14 | 20.3 | 22.32 | 1.135 | 0.813 |
| CBM38 | 0.35 | 0.44 | 0.34 | 0.116 | 0.941 |
| CBM47 | 0.35 | 0.18 | 0.18 | 0.066 | 0.56 |
| CBM48 | 15.74 | 15.52 | 14.16 | 1.365 | 0.904 |
| CBM50 | 17.19 | 15.95 | 13.81 | 1.063 | 0.482 |
| CBM51 | 0.93 | 1.92 | 1.49 | 0.271 | 0.372 |
| CBM54 | 2.33 | 2.17 | 1.60 | 0.26 | 0.545 |
| CBM57 | 0.75 | 2.00 | 0.93 | 0.435 | 0.513 |
| CE1 | 15.92 | 16.24 | 20.04 | 1.018 | 0.193 |
| CE4 | 6.32 | 6.28 | 5.83 | 0.57 | 0.945 |
| CE6 | 10.51 | 7.22 | 7.92 | 0.94 | 0.367 |
| CE7 | 5.01 | 1.88 | 4.18 | 0.669 | 0.133 |
| CE8 | 7.26 | 6.65 | 5.81 | 0.664 | 0.726 |
| CE9 | 14.38 | 15.8 | 15.46 | 2.172 | 0.971 |
| CE10 | 16.13 | 20.33 | 18.73 | 1.781 | 0.687 |
| CE11 | 15.67 | 16.07 | 13.12 | 1.504 | 0.743 |
| CE12 | 2.78 | 3.60 | 4.46 | 0.429 | 0.317 |
| CE14 | 2.23^b^ | 1.11^ab^ | 0.83^a^ | 0.279 | 0.070 |
| CE15 | 2.14 | 2.83 | 2.57 | 0.434 | 0.846 |
| GH1 | 1.24 | 1.57 | 1.42 | 0.188 | 0.819 |
| GH2 | 8.93 | 8.72 | 9.46 | 0.689 | 0.926 |
| GH3 | 9.34 | 10.26 | 9.43 | 0.753 | 0.889 |
| GH4 | 1.29 | 0.72 | 1.02 | 0.13 | 0.206 |
| GH5 | 3.5 | 3.54 | 3.26 | 0.237 | 0.901 |
| GH8 | 0.42 | 0.39 | 0.43 | 0.064 | 0.964 |
| GH9 | 2.23^b^ | 1.17^a^ | 1.60^ab^ | 0.197 | 0.058 |
| GH10 | 0.95 | 2.1 | 1.08 | 0.244 | 0.088 |
| GH12 | 0.23 | 0.27 | 0.28 | 0.062 | 0.944 |
| GH13 | 5.92 | 6.83 | 6.17 | 0.589 | 0.850 |
| GH16 | 1.92 | 1.19 | 1.23 | 0.273 | 0.529 |
| GH18 | 1.28 | 1.57 | 1.11 | 0.161 | 0.555 |
| GH20 | 1.13 | 0.89 | 1.33 | 0.143 | 0.513 |
| GH23 | 2.77^b^ | 1.87^a^ | 3.33^b^ | 0.246 | 0.014 |
| GH25 | 0.95 | 1.24 | 1.35 | 0.11 | 0.348 |
| GH26 | 0.75 | 0.76 | 0.66 | 0.177 | 0.978 |
| GH27 | 0.29 | 0.45 | 0.34 | 0.054 | 0.536 |
| GH28 | 1.05^a^ | 1.70^b^ | 1.81^b^ | 0.141 | 0.027 |
| GH29 | 1.87 | 1.22 | 1.53 | 0.296 | 0.728 |
| GH30 | 0.62 | 0.54 | 0.63 | 0.087 | 0.919 |
| GH31 | 2.65 | 5.94 | 2.75 | 0.947 | 0.307 |
| GH32 | 1.12 | 1.01 | 0.91 | 0.197 | 0.926 |
| GH33 | 1.32 | 1.72 | 1.27 | 0.108 | 0.175 |
| GH35 | 0.81 | 0.57 | 1.39 | 0.211 | 0.302 |
| GH36 | 1.66 | 2.1 | 1.53 | 0.168 | 0.394 |
| GH39 | 1.93^b^ | 0.76^a^ | 1.87^b^ | 0.239 | 0.051 |
| GH43 | 7.3 | 6.81 | 8.04 | 0.355 | 0.421 |
| GH45 | 0.19 | 0.16 | 0.26 | 0.053 | 0.808 |
| GH50 | 0.41 | 0.47 | 0.54 | 0.057 | 0.693 |
| GH51 | 1.74 | 2.38 | 2.08 | 0.188 | 0.434 |
| GH53 | 2.63 | 3.23 | 2.09 | 0.445 | 0.644 |
| GH57 | 1.5 | 0.83 | 0.8 | 0.226 | 0.410 |
| GH65 | 0.29 | 0.19 | 0.25 | 0.04 | 0.658 |
| GH67 | 0.29 | 0.45 | 0.45 | 0.044 | 0.310 |
| GH72 | 1.46^b^ | 1.03^ab^ | 0.88^a^ | 0.113 | 0.065 |
| GH73 | 1.24 | 0.87 | 0.9 | 0.133 | 0.509 |
| GH77 | 2.72 | 3.38 | 1.67 | 0.5 | 0.423 |
| GH78 | 1.63 | 2.02 | 2.2 | 0.193 | 0.529 |
| GH89 | 0.35 | 0.17 | 0.21 | 0.047 | 0.301 |
| GH92 | 2.6 | 2.59 | 3.5 | 0.26 | 0.295 |
| GH94 | 2.06 | 1.79 | 1.35 | 0.16 | 0.194 |
| GH95 | 2.5 | 2.25 | 2.24 | 0.117 | 0.656 |
| GH97 | 3.13^b^ | 1.72^a^ | 3.22^b^ | 0.285 | 0.019 |
| GH99 | 0.4 | 0.54 | 0.21 | 0.108 | 0.514 |
| GH105 | 1.44 | 1.56 | 0.93 | 0.189 | 0.407 |
| GH106 | 1.11^a^ | 0.87^a^ | 1.73^b^ | 0.153 | 0.029 |
| GH109 | 1.65 | 1.14 | 1.66 | 0.151 | 0.307 |
| GH115 | 1.14 | 0.75 | 1.25 | 0.134 | 0.313 |
| GH120 | 0.2 | 0.21 | 0.16 | 0.036 | 0.866 |
| GH127 | 0.84^ab^ | 0.56^a^ | 1.28^b^ | 0.129 | 0.038 |
| GH128 | 0.36 | 0.23 | 0.15 | 0.665 | 0.476 |
| GH130 | 0.7 | 0.78 | 0.68 | 0.054 | 0.758 |
| PL1 | 34.77 | 58.46 | 41.71 | 8.178 | 0.541 |
| PL9 | 13.45 | 7.1 | 5.41 | 1.866 | 0.185 |
| PL10 | 12.79 | 6.75 | 8.48 | 2.267 | 0.600 |
| PL11 | 24.77 | 17.39 | 19.34 | 2.624 | 0.557 |
