# Supplementary material for: Impact of levels of total digestible nutrients on microbiome, enzyme profile and degradation of feeds in buffalo rumen
Source: PLoS One. 2017 Feb 16;12(2):e0172051. doi: 10.1371/journal.pone.0172051 (PMC5313230; doi:10.1371/journal.pone.0172051)
Supplement: S3 Table — (DOC) [file pone.0172051.s003.doc]

S3 Table: Abundance (range) of archeal genera in rumen microbiome of buffaloes

| **Attributes** | **70** | **85** | **100** |
| --- | --- | --- | --- |
| *Methanobrevibacter* | 38.3-43.5 | 17.1-72.01 | 36.7-51.5 |
| *Methanococcus* | 0.89-2.56 | 0.00 | 1.3-6.1 |
| *Methanoculleus* | 6.1-7.4 | 0.00-1.2 | 0.00-10.9 |
| *Methanoplanus* | 9.9-11.1 | 4.6-19.4 | 10.5-15.2 |
| *Methanosarcina* | 0.32-2.1 | 0.35-1.69 | 1.01-3.2 |
| *Methanospirillum* | 0.00-7.14 | 0.00-12.6 | 9.7-12.1 |
| *Methanothermobacter* | 0.30-8.4 | 0.00-12.2 | 0.00-9.7 |
| *Methanoregula* | 2.7-3.6 | 0.00-7.9 | 0.00 |
| *Methanosphaerula* | 0.00-5.75 | 0.00-9.62 | 0.00 |
| *Methanocorpusculum* | 0.00-7.74 | 0.00-8.22 | 0.00 |
